# Freestanding Translucent ZnO–Cellulose Nanocomposite Films for Ultraviolet Sensor Applications

**DOI:** 10.3390/nano12060940

**Published:** 2022-03-12

**Authors:** Hiroaki Komatsu, Yurika Kawamoto, Takashi Ikuno

**Affiliations:** Department of Applied Electronics, Graduate School of Advanced Engineering, Tokyo University of Science, Katsushika, Tokyo 125-8585, Japan; 8121516@ed.tus.ac.jp (H.K.); 8120519@ed.tus.ac.jp (Y.K.)

**Keywords:** cellulose nanofiber, ZnO nanoparticles, nanocomposite, film, flexible, photo response, UV sensors

## Abstract

The rapidly advancing technology of wearable and miniaturized electronics has increased the demand for low-cost high-performance flexible sensors. Herein, the preparation of translucent freestanding films consisting of cellulose nanofibers (CNFs) and ZnO nanoparticles (NPs) via a simple spray coating method is presented. The obtained nanocomposite films were thin (~10 µm) and flexible. The scanning electron microscopy and atomic force microscopy analysis revealed that the nanocomposite film was composed of regions of ZnO NP-modified CNFs and regions of aggregation of ZnO NPs with each other. The electrical conductance of the films was rapidly increased beyond 40 wt.% ZnO and reached up to >50 nA at 60 wt.% ZnO. This was attributed to the increased number of conductive paths formed by the ZnO NPs in the nanocomposite film when a certain threshold was crossed. The ZnO–CNF nanocomposite film exhibited a stable response over on/off cycles of UV light exposure. The responsivity and sensitivity of the nanocomposite film with 60 wt.% ZnO were 36.5 mA/W and 247, respectively. Even when the device was curved (radius of curvature: 3 mm), the response and sensitivity remained high. The developed nanocomposite films are expected to be applied as environmentally friendly flexible UV sensors.

## 1. Introduction

In recent years, electronic waste (e-waste) has been increasing at an alarming rate owing to the mass proliferation of electronic devices with a short lifespan [1,2]. One of the solutions to reduce e-waste is to adopt environmentally friendly natural materials [3], such as chitin [4], silk [5], fibroin [6], and cellulose [7,8], for electronic devices instead of oil-derived polymers. Cellulose is a renewable, biodegradable, and ubiquitous biopolymer that is a key source of sustainable materials on an industrial scale [9]. Cellulose nanofiber (CNF)-based films can be used as substrates for thin-film devices, such as paper-based transparent conductive films [10], flexible nonvolatile memories [11], and flexible antennas [12]. For applications in cellulose-based electronic devices, the ZnO–cellulose composite is one of the most important materials because of its mechanical flexibility, light weight, environmental sustainability, and excellent physical and biochemical functions, such as its ultraviolet (UV) light response [13], piezoelectric effect [14], and antibacterial activity [15].

Recently, low-cost ZnO–cellulose UV sensors have been actively studied for wearable sensor applications. For this purpose, a composite with mechanical flexibility and excellent sensor properties is required. The structures of ZnO–cellulose UV sensors reported thus far can be categorized into three types. The first structure is a composite of ZnO nanostructures and cellulose fibers [13,16]. Although the composite device exhibited a high responsivity (>6 A/W), it was deemed to be rigid because the thick composite pellets were fabricated using the high-pressure method. The excellent sensor properties may be attributed to the uniform dispersion of ZnO nanostructures in high concentrations in the cellulose fiber pellets, leading to the formation of a network of ZnO particles responsible for carrier transport between the electrodes. The second structure is a layered structure, in which ZnO nanostructures such as nanoparticles, nanorods, and nanowires can be grown on the surface of cellulose substrates [17]. This type of device has good mechanical flexibility by virtue of the unique properties of cellulose. However, the responsivities (0.39–1.20 μA/W) were relatively lower than those of the first type of devices. The third structure is a hybrid of the first and second structures [18], in which a composite film of ZnO nanostructures and carboxymethyl cellulose is deposited on a copy paper by screen printing. This type of device has both good responsivity (432 mA/W) and mechanical flexibility.

In addition to the sensor properties and mechanical flexibility, adding features such as optical transparency and/or translucency to UV sensors is important for combination with light-emitting devices and photovoltaic devices [19]. Moreover, if the transparency and/or translucency can be increased, the designability can be improved when sensors are used as an element of wearable devices [20,21]. However, as mentioned above, the conventional three types of devices have neither transparency nor translucency because these devices have thick cellulose fibers that scatter incident light. In addition, the devices are too thick to transmit the incident light through them.

Therefore, in this study, to realize a ZnO–cellulose UV sensor with excellent sensor properties, mechanical flexibility, and optical translucency, we fabricated a novel freestanding ZnO–CNF nanocomposite film. The freestanding film is translucent because the diameters of the ZnO nanoparticles (NPs) and CNFs are smaller than the wavelength of visible light, and the thickness of the film is only approximately 10 μm. Furthermore, the relatively uniform dispersion of ZnO NPs and CNFs ensures conductive paths in the film. The film is mechanically flexible because it is composed of a matrix of CNFs.

## 2. Materials and Methods

Typically, an aqueous solution of bamboo-derived CNFs (Bamboo #C, Chuetsu Pulp & Paper Co., Ltd., Toyama, Japan) disintegrated by the aqueous counter collision (ACC) method [22] and ZnO NPs (average diameter = 25 nm; FINEX-50, Sakai Chemical Industry Co., Ltd., Osaka, Japan) were mixed using an ultrasonic homogenizer (FS300N, Shenzhen XinzhiBang Inst &Eqpt. Co., Ltd., China) at 300 W output power for 2 min. The resulting dispersion was then poured into the solution reservoir of a custom-made spraying machine. Soda–lime glass plates (thickness: 1 mm; Tokyo Glass Co., Ltd., Japan) were cleaned with acetone, ethanol, and deionized water. The glass plates were fixed on the surface of a hot plate (HP-1SA, AS ONE Corporation, Osaka, Japan) and heated in air at 110 °C. The ZnO/CNF dispersion was sprayed onto the heated glass plate for approximately 10 min. Finally, the dried film was carefully peeled off. In this manner, ZnO–CNF nanocomposite films with different ZnO NP contents were fabricated.

The optical properties and crystallinity of the resultant films were investigated using UV–visible spectroscopy (V-770, JASCO, Tokyo, Japan) and X-ray diffraction (XRD) (RINT2200, Rigaku, Tokyo, Japan), respectively. The surface morphologies and elemental mapping were characterized using atomic force microscopy (AFM) (SPA-400, Hitachi, Tokyo, Japan) and field-emission scanning electron microscopy (SEM) with energy-dispersive X-ray spectroscopy (EDX) (SUPRA 40, Carl Zeiss, Jena, Germany). To measure the electrical transport properties, a voltage was applied on the top and bottom surfaces of the films. Patterned gold (Au) thin films were deposited on the films using a vacuum evaporator (VPC-410, ULVAC, Kanagawa, Japan). A source meter (2612A, Keithley, OH, USA) and a probe station were used for the current–voltage (*I*–*V*) measurements. The photoconductivity was measured under UV light (λ = 365 nm) illumination using an LED light at a constant voltage of 1.2 V. The distance between the LED light and the films was approximately 2 cm, and the radiation power was 11.1 mW/cm^2^.

## 3. Results and Discussion

### 3.1. Characterization of Freestanding ZnO–CNF Nanocomposite Films

**Figure 1:**
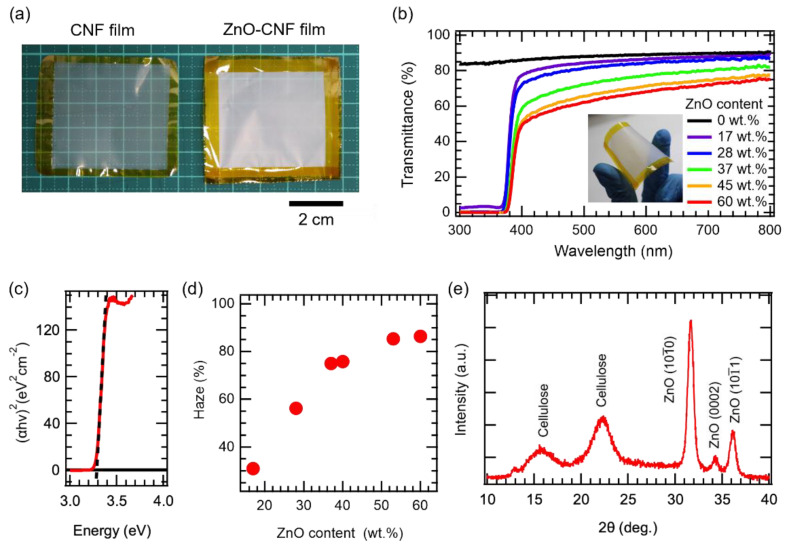
(**a**) Digital photographs and (**b**) optical transmittance spectra of the CNF film without ZnO NPs and ZnO–CNF nanocomposite films. Inset: digital photograph of a curved freestanding film with 37wt.% ZnO. (**c**) Tauc plot of the nanocomposite film with 17 wt.% ZnO NPs. (**d**) Haze values of the nanocomposite films as a function of the ZnO content. (**e**) Typical XRD pattern of the nanocomposite films (ZnO content: 17 wt.%).

Figure 1a shows the photographs of the fabricated CNF film (without ZnO) and nanocomposite film (17 wt.% ZnO). The average thickness of the films was approximately 10 μm. The nanocomposite films were found to be flexible, as shown in the inset of Figure 1b. On visual inspection, the ZnO–CNF nanocomposite films were found to be translucent with transparency lower than that of the CNF film without ZnO NPs. Figure 1b shows the optical transmittance of each film as a function of the wavelength. The transmittance of the CNF film without ZnO NPs exceeded 80% in the visible region (wavelength range: 400–800 nm). The transmittance of the ZnO–CNF nanocomposite films, however, decreased with increasing ZnO content. Nevertheless, the transmittance exceeded 50% even when the ZnO content was 60 wt.%, indicating the translucency of the ZnO–CNF nanocomposite films. The sharp decrease in transmittance below 370 nm was due to the interband photon absorption of ZnO. Figure 1c shows the Tauc plot [23] of the nanocomposite film with 17 wt.% ZnO. By extrapolating a straight line to the curve, the optical band gap was estimated to be 3.3 eV, which is consistent with the band gap of ZnO [24].

Figure 1d shows the haze values [25] as a function of the ZnO content in the films. The haze value of the CNF film without ZnO NPs was ~22.4%. The haze value of the ZnO–CNF nanocomposite films was found to increase with the ZnO content and saturated at 85% at a ZnO content of more than 50 wt.%. Even though the thickness of CNFs and the diameter of ZnO NPs were sufficiently smaller than the wavelength of the incident light, the haze values were high. The high haze values imply the existence of aggregations of ZnO NPs, which play the role of light scattering.

Figure 1e shows the XRD pattern of the ZnO–CNF nanocomposite films containing sharp peaks at 31.66°, 34.30°, and 36.12° and broad peaks at 15.66° and 22.36°. The sharp peaks were attributed to the ZnO (JCPDS Card No: 36-1451), whereas the broad peaks originated from cellulose, corresponding to cellulose Iα (14.85°, 16.66°, and 22.98°; JCPDS Card No: 56-1719) and cellulose Iβ (14.26°, 16.77°, and 21.80°; JCPDS Card No: 56-1718).

**Figure 2:**
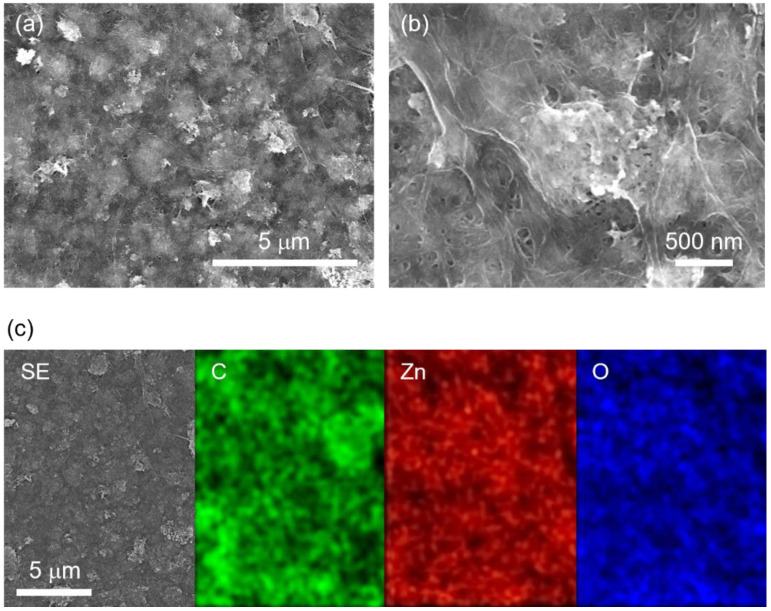
SEM images of (**a**) low and (**b**) high magnification of the nanocomposite film with 45 wt.% ZnO. (**c**) Secondary electron image and EDX elemental mappings of C, Zn, and O of nanocomposite film with 45 wt.% ZnO.

Next, the morphologies and elemental mappings of the film were characterized using SEM and EDX. Figure 2a shows the low-magnification SEM image of the film with 45 wt.% ZnO. The contrast of the image was not uniform. The difference in the contrast might be caused by the difference in the concentration of ZnO NPs in the film. To investigate in detail, we observed a high-magnification SEM image, as shown in Figure 2b. CNFs were entangled, and the contrast between black and white was gradually changed rather than separated clearly. Figure 2c shows the secondary electron image and elemental mappings of C, Zn, and O. C and Zn indicate distributions of CNFs and ZnO NPs, respectively. The O elemental mapping corresponds to both of them. From the C elemental mapping, CNF-rich and CNF-poor regions were found in the film. The Zn elemental mapping showed that Zn was distributed throughout the entire area, although there were some empty spots, indicating that ZnO NPs were distributed over the entire film. From the results of SEM and EDX, neither CNFs nor ZnO NPs themselves were aggregated in the film.

**Figure 3:**
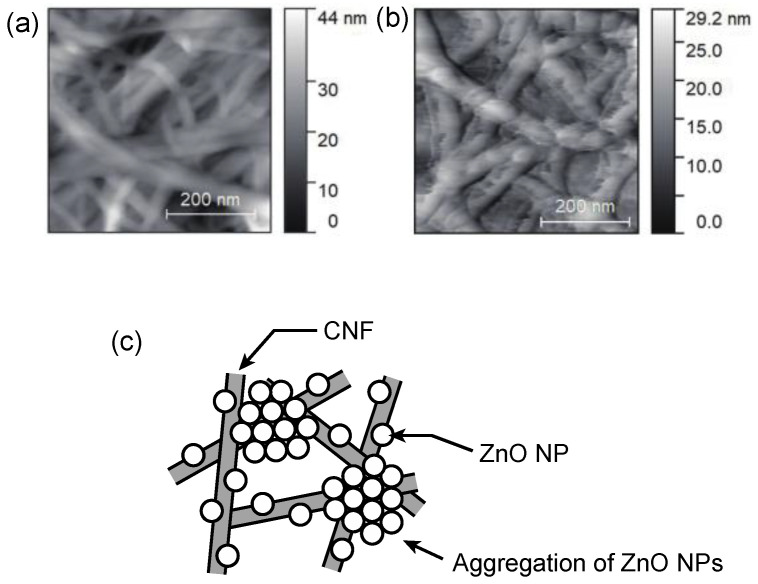
AFM image of (**a**) CNF film without ZnO NPs and (**b**) nanocomposite film with 40 wt.% ZnO. (**c**) Schematic illustration of the conceivable local structure of our film.

To understand the local structure of ZnO–CNF interfaces, AFM observation was carried out. Figure 3a,b show the AFM surface images of the CNF film without ZnO NPs and the nanocomposite film with 40 wt.% ZnO NPs, respectively. The CNF film without ZnO NPs exhibited a morphology with entangled nanofibers. Although the nanocomposite film also had a similar morphology, the nanofibers were relatively thicker. In addition, rough structures were observed on the surface of the nanofibers, suggesting the presence of ZnO NPs. The selective adsorption of ZnO NPs on CNFs occurred because of the electrostatic attraction between Zn atoms of the ZnO (001) and O atoms of the CNFs, as revealed by density functional theory (DFT) calculations [26].

In practice, there may be not only selective adsorption of ZnO NPs on CNF surfaces but also aggregations of ZnO NPs themselves, as shown in Figure 2. Therefore, based on the results of SEM, EDX, and AFM, our films are considered to be composed of regions of ZnO NP-modified CNFs and regions of aggregation of ZnO NPs with each other, as shown in Figure 3c. The film exhibited translucency, even though aggregation regions exist, possibly because the thickness of the film is thin enough for light transmission. To analyze the details of the structure, further analysis such as tomography is needed.

### 3.2. Electrical Properties of ZnO–CNF Nanocomposite Films

**Figure 4:**
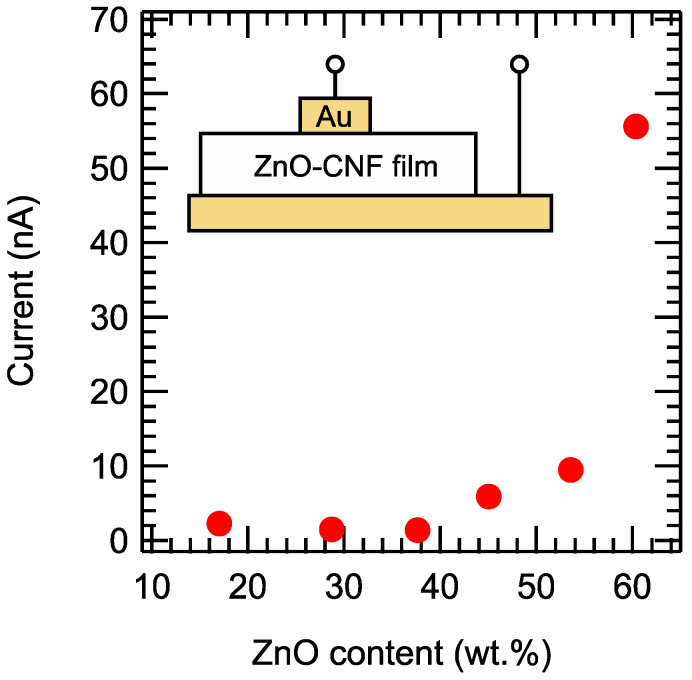
Current through the ZnO–CNF nanocomposite films as a function of the ZnO content at a constant voltage of 1.2 V. Inset: Schematic illustration of the measurement setup.

To examine the electronic properties of the ZnO–CNF nanocomposite films, the current through the films was measured by applying a constant voltage of 1.2 V between the top and bottom surfaces, as shown in the inset of Figure 4. The surface area of the top electrode was 4 mm^2^. Figure 4 shows the variation in the current as a function of the ZnO content. When the ZnO content was less than 40 wt.%, the current value was only 1–3 nA. However, the current value increased beyond 40 wt.%. A current value greater than 50 nA was achieved at 60 wt.%. The maximum error range of the current was ±5.8 nA. Thus, there is a threshold value at which carrier transport is activated. This behavior could be explained by percolation theory [27]. The film sample was a nanocomposite based on insulating CNFs and semiconducting ZnO NPs. When the number of semiconducting NPs responsible for carrier transport crossed a threshold value, conductive paths were formed between the electrodes [28]. This significant increase in the current value at a certain threshold value was also confirmed by a Monte Carlo simulation (Figure S1).

**Figure 5:**
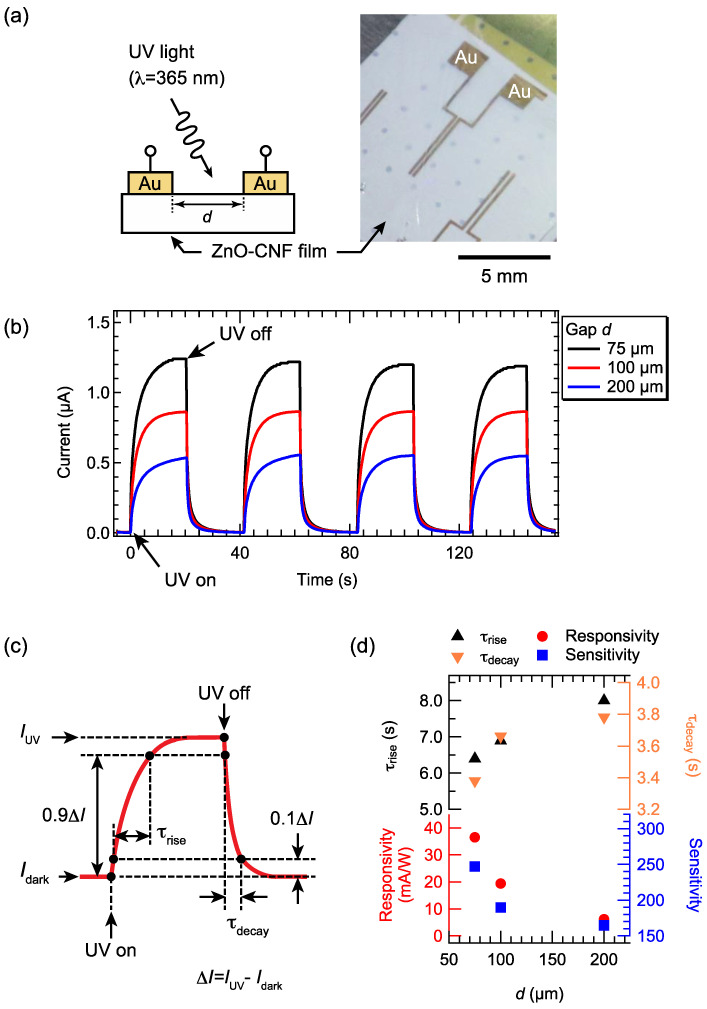
(**a**) Schematic illustration and photograph of the measurement setup. The suction holes of the *I–V* prober are visible through the ZnO–CNF film because the film is translucent. (**b**) Time evolution of UV light-induced photocurrent for the films with various ZnO contents at the applied voltage of 1.2 V. (**c**) Schematic illustration of one on/off UV cycle indicating the definition of *I*_UV_, *I*_dark_, Δ*I*, rise time (τ_rise_), and decay time (τ_decay_). (**d**) τ_rise_, τ_decay_, responsivity, and sensitivity as a function of the electrode gap, which were estimated from Figure 5b.

Next, we investigated the UV sensor properties of the ZnO–CNF nanocomposite film with 60 wt.% ZnO NPs. A UV light-induced photocurrent through the film was measured at an applied voltage of 1.2 V between a pair of patterned electrodes, as shown in Figure 5a. The lengths of the electrode legs were 4 mm. We fabricated various devices, in which gaps of the pair of electrodes were 75, 100, and 200 μm. Intermittent UV light with a period of approximately 20 s was irradiated onto the surface to observe the photocurrent. Figure 5b shows the time evolution of the photocurrent as a function of the gap. Without UV light irradiation, the dark currents, *I*_dark_, for all devices were approximately 3.4–4.9 nA. By irradiating UV light, the currents were steeply increased. By increasing the gap, the maximum photocurrent, *I*_UV_, was decreased. From Figure 5b, we estimated the sensor properties such as responsivity, sensitivity, and response times [29]. These parameters were defined, as shown in Figure 5c. The responsivity, *R*, was estimated from *I*_dark_, *I*_UV_, and the optical power of the UV source, *P*_op_, based on the equation: *R* = (*I*_UV_ − *I*_dark_)/*P*_op_. The numerator in the above equation is called the response current, Δ*I*. The sensitivity, *S*, was defined as the equation: *S* = *I*_UV_/*I*_dark_. The switching response of the UV sensors was also determined to characterize how fast they respond during and after UV light irradiation. The rise time, τ_rise_, was determined by the time required to shift from 10 to 90% of Δ*I*, while the decay time, τ_decay_, corresponds to the time required to decrease from 90 to 10% of Δ*I* when the UV light was turned off.

Figure 5d shows the parameters as a function of the gap. The responsivities and sensitivities decreased with increasing gap. The maximum values of *R* and *S* were 36.5 mA/W and 247, respectively, for the device with a gap of 75 μm. Compared to previously reported devices in which ZnO nanostructures were deposited on Whatman paper [18], our device showed three orders of magnitude larger responsivity, with similar sensitivity. Moreover, compared to previously reported devices in which a ZnO/CMC composite layer was deposited on copy paper, our device showed 18 times larger sensitivity.

The higher responsivity and sensitivity of our nanocomposite films compared with those reported previously could be attributed to the short interelectrode distance. The interelectrode distance of 75 μm, which is shorter than that in previous studies [13,18,19], may have reduced the scattering of photoexcited carriers at the ZnO NP interface during their transport.

We estimated τ_rise_ and τ_decay_ of our nanocomposite films, as shown in Figure 5d. τ_rise_ and τ_decay_ were decreased by decreasing the interelectrode gaps. For *d* = 75 μm, τ_rise_ and τ_decay_ were 6.4 s and 3.4 s, respectively. The response times were smaller than those reported for ZnO–cellulose UV sensors in previous studies. For instance, the ZnO/CMC UV sensor, which has high responsivity, exhibited τ_rise_ and τ_decay_ of 8.2 s and 22.0 s, respectively. Our device showed short response times because the carrier transport paths made of ZnO NP arrays might be effectively formed in the film as ZnO NPs with diameters similar to those of CNFs, which might be selectively adsorbed on the CNF surfaces.

**Figure 6:**
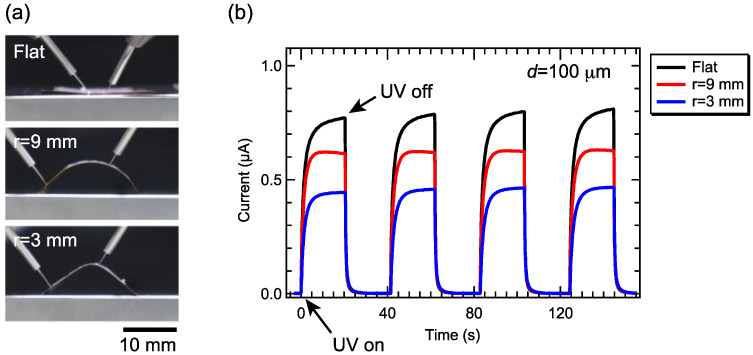
(**a**) Photographs of ZnO–CNF UV sensors in bending states. (**b**) Time evolution of UV light-induced photocurrent for the films with various ZnO contents at the applied voltage of 1.2 V.

Next, we placed the device (ZnO–CNF nanocomposite film with 60 wt.% ZnO NPs) on a Cu plate, and the changes in the photo response of the device were investigated during bending with curvature radii of 3 and 9 mm. Figure 6a shows cross-sectional photographs of the device placed on the Cu plate with and without bending. Figure 6b shows the time evolution of the photo response with intermittent UV irradiation. With decreasing curvature, the values of *I*_UV_ were found to decrease. *I*_UV_ for *r* = 3 mm was 60% of *I*_UV_ for the flat device. This can be explained both by the increase in resistance and the decrease in the incident light intensity due to bending. In the previous report, the responsivity of ZnO nanorod UV sensors grown on paper with bending curvatures of 15 cm was approximately 10% of that for the device without bending. Compared to the previous report, our device showed less change; even the curvature is smaller than the previous one.

## 4. Conclusions

In this study, freestanding ZnO–CNF nanocomposite films were prepared via a spraying method. The films were translucent in the visible region because of their thinness. The responsivity and sensitivity of the films were higher than those of the reported ZnO/cellulose UV sensors, which might be attributed to the shortening of the interelectrode distance and formation of effective carrier paths in the film.

As these freestanding nanocomposite films have a low surface roughness, it is expected that interdigitated finger electrodes with a narrow interelectrode distance can be fabricated via photolithography in the future. We believe that such a structure would achieve even higher responsivity and sensitivity. Additionally, these films are also expected to show antibacterial activity [15] and may be applied to biodegradable electronic devices because of their environmentally friendly component materials.

## Data Availability

Not applicable.

## Supplementary Materials

The following supporting information can be downloaded at: https://www.mdpi.com/article/10.3390/nano12060940/s1, Figure S1: (a) Simulation model for carrier transport in CNF films with ZnO NPs based on Monte Carlo method. (b) Simulation result of ZnO NPs Contents vs. Effective current.
