# Supplementary material for: Freestanding Translucent ZnO–Cellulose Nanocomposite Films for Ultraviolet Sensor Applications
Source: Nanomaterials (Basel). 2022 Mar 12;12(6):940. doi: 10.3390/nano12060940 (PMC8954166; doi:10.3390/nano12060940)
Supplement: Supplementary file 1 [file nanomaterials-12-00940-s001.zip › nanomaterials-1606176-supplementary.pdf]

nanomaterials

Supplementary Information

Freestanding Translucent ZnO–Cellulose Nanocomposite Films for Ultraviolet Sensor Applications

Hiroaki Komatsu, Yurika Kawamoto and Takashi Ikuno *

Department of Applied Electronics, Graduate School of Advanced Engineering, Tokyo University of Science, Katsushika, Tokyo 125-8585, Japan; 8121516@ed.tus.ac.jp (H.K.); 8120519@ed.tus.ac.jp (Y.K.)

* Correspondence: tikuno@rs.tus.ac.jp

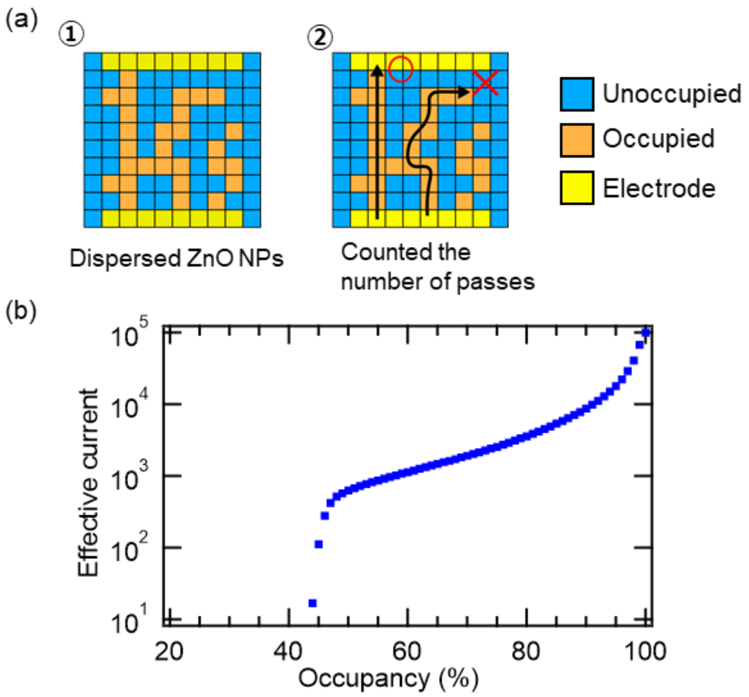

Figure S1. (**a**) Simulation model for carrier transport in CNF films with ZnO NPs based on Monte Carlo method. (**b**)Simulation result of ZnO NPs Contents vs Effective current.
